# Graphene–aluminum nitride NEMS resonant infrared detector

**DOI:** 10.1038/micronano.2016.26

**Published:** 2016-06-20

**Authors:** Zhenyun Qian, Yu Hui, Fangze Liu, Sungho Kang, Swastik Kar, Matteo Rinaldi

**Affiliations:** 1 Department of Electrical and Computer Engineering, Northeastern University, Boston, MA 02115, USA; 2 Department of Physics, Northeastern University, Boston, MA 02115, USA

**Keywords:** aluminum nitride, graphene, infrared detector, MEMS, NEMS, piezoelectric, resonant sensor

## Abstract

The use of micro-/nanoelectromechanical resonators for the room temperature detection of electromagnetic radiation at infrared frequencies has recently been investigated, showing thermal detection capabilities that could potentially outperform conventional microbolometers. The scaling of the device thickness in the nanometer range and the achievement of high infrared absorption in such a subwavelength thickness, without sacrificing the electromechanical performance, are the two key challenges for the implementation of fast, high-resolution micro-/nanoelectromechanical resonant infrared detectors. In this paper, we show that by using a virtually massless, high-electrical-conductivity, and transparent graphene electrode, floating at the van der Waals separation of a few angstroms from a piezoelectric aluminum nitride nanoplate, it is possible to implement ultrathin (460 nm) piezoelectric nanomechanical resonant structures with improved electromechanical performance (>50% improved frequency×quality factor) and infrared detection capabilities (>100× improved infrared absorptance) compared with metal-electrode counterparts, despite their reduced volumes. The intrinsic infrared absorption capabilities of a submicron thin graphene–aluminum nitride plate backed with a metal electrode are investigated for the first time and exploited for the first experimental demonstration of a piezoelectric nanoelectromechanical resonant thermal detector with enhanced infrared absorptance in a reduced volume. Moreover, the combination of electromagnetic and piezoelectric resonances provided by the same graphene–aluminum nitride-metal stack allows the proposed device to selectively detect short-wavelength infrared radiation (by tailoring the thickness of aluminum nitride) with unprecedented electromechanical performance and thermal capabilities. These attributes potentially lead to the development of uncooled infrared detectors suitable for the implementation of high performance, miniaturized and power-efficient multispectral infrared imaging systems.

## Introduction

The recent advances in miniaturization, power efficiency and cost reduction of sensor technologies are paving the way for the development of highly distributed wireless sensor networks capable of gathering a large amount of information from the environment, with high accuracy and reliability, through the exploitation of multiple sensing and wireless communication functionalities. This trend towards sensor fusion has markedly increased the demand for a new technology platform capable of delivering multiple sensing and wireless communication functionalities in a small footprint. In this context, micro- and nanoelectromechanical systems (MEMS/NEMS) may provide a huge impact, since they can be used for the implementation of miniaturized, high-performance sensors and wireless communication devices fully compatible with standard integrated circuitry. The utilization of NEMS has been explored in many applications, spanning from semiconductor-based technology^1^ to fundamental science^2^. In particular, NEMS resonators have been employed successfully as ultrasensitive detectors for sensing mass^3^, fluid flow^4^, chemicals^5–7^, and biological agents^8^, just to mention a few. The fundamental advantage of NEMS resonant sensors over other technologies is related to their unique combination of extremely high sensitivity to external perturbations (due to their highly reduced dimensions) and ultralow noise performance (due to the intrinsically high-quality factor, *Q*, of such resonant system). Moreover, the output variable of such resonant sensors is a frequency, which is one of the physical quantities that can be monitored with the highest accuracy and converted to digital form by measuring zero-crossings.

In recent years, the use of piezoelectric micro- and nanoscale electromechanical resonators for room temperature detection of infrared (IR) radiation has attracted a great deal of attention^9–11^ due to the intrinsically high temperature sensitivity (Temperature Coefficient of Frequency, TCF: aluminum nitride (AlN), approximately −25 p.p.m. per K (Refs. 12, 13); gallium nitride, approximately −18 p.p.m. per K (Ref. 14); Y-cut quartz ~90 p.p.m. per K (Ref. 10); lithium niobate, approximately −80 p.p.m. per K (Ref. 15), and excellent thermal isolation (~10^6^ K W^−1^) offered by such suspended resonant micro/nanostructures^16^. In particular, thermal detection capabilities comparable to or even outperforming conventional microbolometers have been demonstrated^17,18^.

The fundamental challenges for the implementation of fast, high-resolution piezoelectric MEMS/NEMS resonant IR detectors are the scaling of the device thickness in the nanoscale range and the simultaneous achievement of high IR absorption in such a deep subwavelength thickness, without sacrificing the electromechanical performance. In fact, the scaling of the device thickness inevitably yields a lower device thermal mass (faster response time) and higher thermal sensitivity (higher thermal resistance). In this context, AlN offers some unique advantages over other piezoelectric materials: ultrathin (tens to hundreds of nanometers) and high-quality AlN films can be directly deposited onto silicon substrates by a low-temperature sputtering process. However, the physical and electrical properties of the device metal electrodes fundamentally limit the volume scaling of such resonators. In fact, the bulky metal electrodes attached to the piezoelectric resonant body of the device have been confirmed as a source of mechanical loading and energy loss^19,20^, which reduces the resonance frequency, *f*_0_, and the quality factor, *Q*, of the device. Although metal electrodes much thinner than the piezoelectric plate are highly desirable, owing to the requirement of high-electrical conductivity, the thickness of such metal layers can hardly be scaled proportionally to that of the piezoelectric vibrating body of the device using current microfabrication techniques. Furthermore, these conventional metal electrodes make the resonant structure highly reflective at IR wavelengths, requiring the attachment of an additional IR absorber on top of the resonator to achieve moderate absorption (>10%) in the IR range^21–23^. The electrical and mechanical loading effects associated with such relatively bulky IR absorbing material (or material stack) attached to the vibrating body of the micro/nanostructure inevitably deteriorate the electromechanical and thermal properties of the resonator (and hence the detection capability and power efficiency of the IR sensor)^22^.

To address these issues, in this work, a monolayer graphene sheet is utilized, in lieu of a conventional metal film, as a top electrically floating electrode in the lateral field scheme^24^ used to excite a high-frequency (~307 MHz) lateral-extensional mode vibration in an AlN piezoelectric nanoplate. Graphene, an atomically thin sheet of carbon, the thinnest known material with high-electrical conductivity and transparency, has been utilized as an electrode in a number of applications^25–30^, including optoelectronics, nanoelectronics, and energy devices. Unlike metal electrodes that form chemical bonds with underlying substrates, graphene remains attached through weak van der Waals interactions and virtually ‘floats’ over the AlN nanoplate with minimal mechanical interactions, which minimizes energy dissipation due to electrode damping and interfacial strain (high *Q*)^31^. Preliminary results outlining the general idea of using the graphene–aluminum nitride (G–AlN) nanoplate resonators for IR sensing were recently reported in a symposium by our group^32^. In this work, by means of in-depth studies and extensive experimental characterizations, we demonstrate that the virtually massless graphene electrode not only boosts the electromechanical performance of the resonator, but its transparent nature also enables effective IR absorption in the ultrathin piezoelectric resonant body of the device, making such G–AlN nanoplate resonators promising candidates for the development of ultrafast and high-resolution NEMS resonant IR detectors.

## Materials and methods

**Figure 1:**
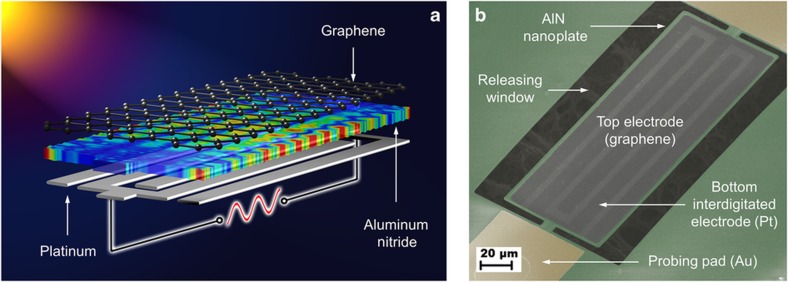
Graphene electrode for NEMS resonant IR detectors. (**a**) Schematic illustration in layer view of a G–AlN NEMS resonator vibrating in its contour-extensional mode. The top electrode, which is critical in confining the electric field within the AlN nanoplate membrane, is fabricated using mechanically transferred, CVD-synthesized graphene. The color map displayed on the AlN layer is created by 3D FEM simulation, which indicates the locations of maximum (red) and minimum (blue) mechanical displacement of the contour-extensional mode in the AlN nanoplate. (**b**) A false-colored tilted-SEM image of a fabricated G–AlN NEMS resonant IR detector. The device is 75-μm wide and 200-μm long.

The proposed G–AlN NEMS resonant IR detector is composed of a freestanding, ultrathin (460 nm) AlN nanoplate supported mechanically at two ends, as shown in Figures 1a and b. A high-frequency bulk acoustic mode of vibration is excited into the AlN nanoplate via a 100-nm thick bottom platinum (Pt) interdigitated electrode (IDE) and a top electrically floating graphene electrode, which replaces the top metal electrode conventionally employed to confine the excitation field across the thickness of the piezoelectric nanoplate^12,33^. Whereas the conventionally employed top metal electrode makes the surface of the device highly reflective at IR wavelengths, the transparent nature of the atomically thin graphene electrode used in this work allows confinement of the impinging IR radiation within the ultrathin resonant structure. In fact, a Fabry–Perot resonance is induced in the cavity formed by the G–AlN-Pt stack when the wavelength, *λ*, of the impinging IR radiation is approximately equal to 2*nt*/(*m*+1/2), where *n* is the refractive index of AlN, *t* is the thickness of the AlN plate, and *m* is the order of the resonance mode.

Moreover, a high-order contour-extensional mechanical mode of vibration is excited through the equivalent *d*_31_ piezoelectric coefficient of AlN when the frequency of the alternating current (AC) signal applied to the bottom IDE coincides with the natural mechanical resonance frequency, *f*_0_, of the structure. Given the equivalent mass density, *ρ*_eq_, and Young’s modulus, *E*_eq_, of the material stack (AlN and electrodes) that forms the resonator, the center frequency, *f*_0_, of this laterally vibrating mechanical resonator is univocally set by the pitch, *W*_0_, of the bottom IDE^13^. The resonance frequency of the device can be expressed as Equation (1) (Ref. 34). The pitch size, *W*_0_, was set to be 15 μm in this work.

(1)f0=12W0Eeqρeq

When an incident IR beam impinges on the top surface of the device and gets absorbed by the G–AlN–Pt stack, a large and fast temperature increase, Δ*T*, of the device is incurred owing to the excellent thermal isolation and extremely low thermal mass of the freestanding nanomechanical structure. This IR-induced temperature increase results in a shift in the mechanical resonance frequency of the resonator due to the intrinsically large temperature coefficient of frequency (TCF) of the device^12^. Therefore, the incident IR power can be readily detected by monitoring the resonance frequency of the device. The responsivity of the resonant IR sensor can be expressed as in Equation (2), where *η* and *R*_th_ are the absorption coefficient and thermal resistance of the NEMS resonator, respectively.

(2)Rs=η⋅Rth⋅TCF⋅f0

**Figure 2:**
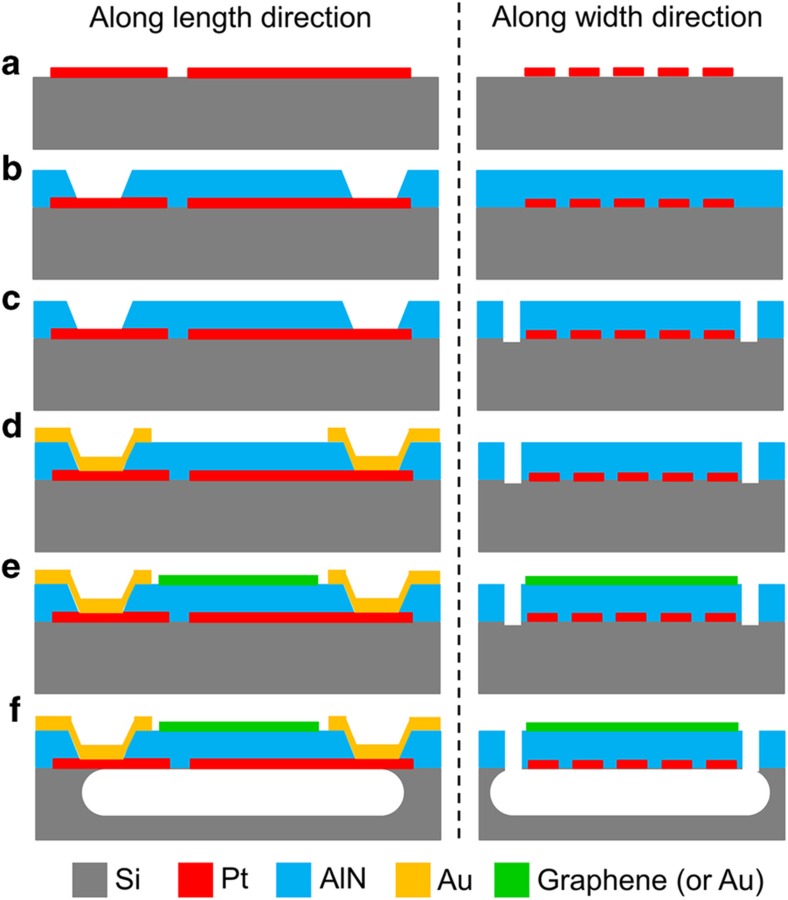
Microfabrication process of G–AlN and reference devices. (**a**) Mask 1—sputter deposition and lift-off of Pt bottom electrode; (**b**) Mask 2—sputter deposition of AlN, wet etch to open vias; (**c**) Mask 3—dry etch to define device lateral dimensions; (**d**) Mask 4—sputter deposition and lift-off of top Au probing pads; (**e**) Mask 5—top electrode synthesis (graphene transfer or gold deposition) and patterning; (**f**) XeF_2_ dry release of the AlN resonators (no mask required). Steps **a** to **d** were processed at the wafer level, with **e** and **f** at the die level.

A combination of top–down microfabrication techniques (5 masks) and bottom–up CVD growth for graphene was employed to fabricate the G–AlN NEMS resonators of this work (Figure 2). PMMA was used as mask material to pattern the graphene layer and protect it during the resonator release step in XeF_2_: A yield of ~90% was achieved for G–AlN devices. High-quality graphene was maintained throughout the fabrication process as confirmed by a Raman spectrum taken after the release of the G–AlN resonators (Supplementary Information). Conventional AlN NEMS resonators, based on the same core design but employing a 100-nm-thick gold top electrically floating electrode^33^ instead of the virtually massless and electrically floating graphene electrode (gold is typically used as the top metal electrode in NEMS resonant sensors because it can be easily functionalized with thiolated ligands^5^), were fabricated simultaneously on the same substrate to be used as reference devices. In total, 13 G–AlN NEMS resonators and 15 reference devices (conventional AlN NEMS resonators) were successfully fabricated (on the same wafer) and tested in this work.

The fabricated G–AlN NEMS resonators and reference devices were tested at room temperature and atmospheric pressure in a radio frequency (RF) probe station, and their electrical responses (one-port scattering parameter, *S*_11_) were measured using an Agilent E5071C (Agilent Technologies, Santa Clara, CA, USA) network analyzer after performing a short-open-load calibration on a standard substrate. The electromechanical performance of the devices was extracted by the modified Butterworth-Van Dyke (MBVD) model fitting of the admittance curves (*Y*_11_) obtained by direct conversion from the measured *S*_11_. The IR absorption spectra of the material stacks forming the G–AlN and reference devices were extracted from the reflectance spectra measured by Fourier transform infrared spectroscopy (FTIR) in the 1–16 μm spectral range (assuming the transmittance to be ~0). An optical measurement setup including a 5-μm quantum cascade laser (QCL) from Pendar Technologies, a chopper and a focusing lens were used to characterize the IR detection capabilities of the G–AlN and the reference devices.

## Results

### Electromechanical performance

**Figure 3:**
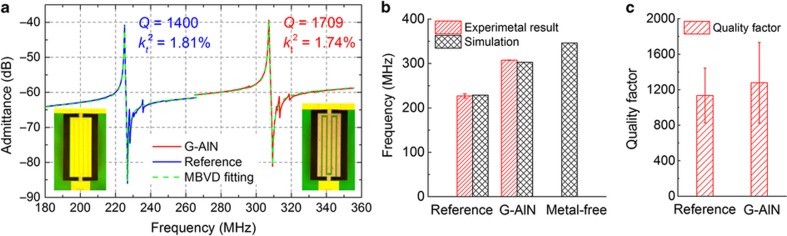
Electromechanical performance of the fabricated G–AlN and reference devices. (**a**) Measured admittance curves and MBVD fitting of a G–AlN resonator and a reference device. The inset shows from left to right micrographs of a reference device with 100-nm thick Au top electrode and a graphene-electrode device. (**b**) Comparison between the measured operating frequencies of the fabricated 13 G–AlN NEMS resonators and the 15 reference devices and their FEM simulation results including an ideal metal-free resonator with the same *W*_0_. (**c**) *Q* factor comparison.

The measured admittance of a G–AlN resonator and a reference device with a 100-nm thick Au top-electrode are shown in Figure 3a. A significant enhancement in resonance frequency is obtained (*f*_0_~307.3 MHz) in the graphene-electrode device compared with the reference device (*f*_0_~225.1 MHz) without any loss of resonance amplitude, which outlines the superiority of using graphene as a massless electrode in piezoelectric NEMS resonators. MBVD model fitting (Figure 3a) was used to extract the electromechanical performance (for example, *Q* and *k*_*t*_^2^) of all fabricated G–AlN and reference devices.

The virtually massless nature of the graphene electrode was experimentally verified by comparing the measured operating frequencies of 13 fabricated G–AlN NEMS resonators with the FEM-simulated frequency of an ideal resonator, based on the same geometry but using ideal conductive boundaries instead of the electrodes. In the simulation tool, the entire top surface of the AlN membrane is treated as a perfectly conducting equipotential sheet that provides the necessary electronic confinement of the RF field within the piezoelectric layer, without adding any mass (Supplementary Information). As indicated in Equation (1), for a given IDE pitch and AlN thickness, the thicknesses and properties of electrodes directly affect the resonance frequency of the device as they set the equivalent sound velocity (mass density, *ρ*_eq_, and Young’s modulus, *E*_eq_) of the material stack forming the resonator. By using graphene as electrode material, the contribution of the electrode to the equivalent density and Young’s modulus of the resonator is eliminated, due to the two-dimensional nature of graphene and its minimal interaction with underlying AlN. Therefore, the sound velocity of the graphene NEMS resonators is maximized, and the devices operate at their theoretical ‘unloaded’ frequency limits. Figure 3b shows that the fabricated G–AlN resonators demonstrate an average vibration frequency that is 35.5% higher than that of the reference devices and closely matches the value predicted by FEM simulation for an ideal resonator. The simulation result of a metal-free device (ultimate scaling limit) in which both top and bottom electrodes are replaced by ideal conductive boundaries has also been included, showing that despite the use of a conventional Pt bottom IDE, the introduction of the graphene top-electrode is sufficient to achieve operating frequencies approaching the metal-free limit. It is also interesting to note that the frequency variation (standard deviation (s.d.)=0.3 MHz) in the experimental data obtained from 13 G–AlN resonators is too small to show in Figure 3b. In particular, the s.d. of the vibrating frequency was found to be much smaller for the graphene-electrode devices compared with the reference devices with a 100-nm Au top electrode (0.3 MHz versus 4.8 MHz), which suggests the great advantage of using graphene as a massless electrode to drastically reduce the variance in the device operating frequency due to the fabrication process variations.

The effectiveness of the virtually massless and strainless graphene electrode in minimizing the energy dissipation due to electrode damping and interfacial strain was experimentally verified by comparing the electromechanical performance of 13 fabricated G–AlN NEMS resonators with that of 15 reference devices. Figure 3c shows the comparison between the average values of quality factor of the fabricated 13 G–AlN resonators and the 15 references devices, demonstrating that the use of the virtually massless and strainless graphene electrode not only boosts the operating frequency of the resonator but also enables the achievement of higher electromechanical performance (12.6% improved *Q*, 52.6% improved *f·Q*). It is worth noting that, although the graphene electrode essentially floats over the AlN nanoplate with minimal mechanical interactions, a high average electromechanical coupling coefficient, *k*_*t*_^2^_G–AlN_=1.7% was measured for G–AlN resonators, which is comparable to that of reference devices employing metal electrodes (*k*_*t*_^2^_AlN_=1.8%).

### IR detection

**Figure 4:**
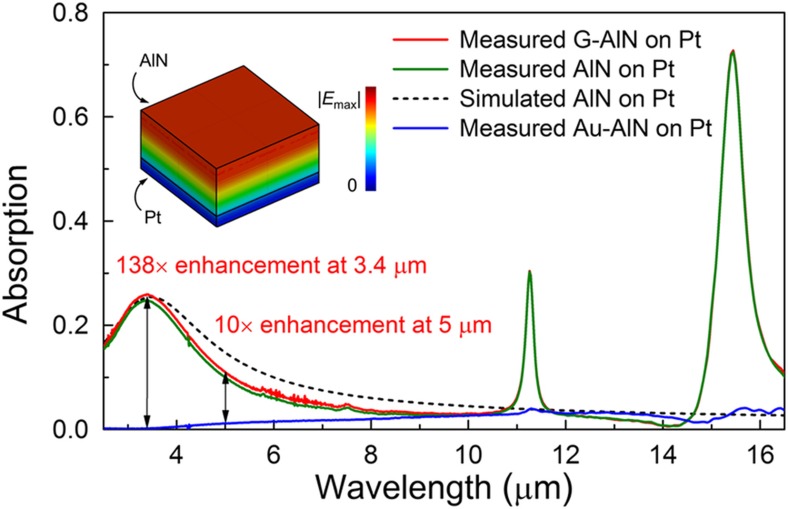
Measured and simulated IR absorption spectra. Three solid lines are the spectra for different materials on top of 460 nm AlN and 100 nm Pt. The dashed line is the simulated spectrum for only the AlN–Pt stack. The inset shows the simulated electric field distribution of the fundamental mode of the Fabry–Perot resonance at 3.4 μm.

We next investigate the IR detection capabilities of the fabricated G–AlN resonators and compare them with those of the reference devices employing a 100-nm thick Au top electrode. Whereas the top metal electrode, conventionally employed for piezoelectric actuation, makes the resonant structure highly reflective at IR wavelengths, the transparent nature of the atomically thin graphene electrode allows exploitation of the intrinsic IR absorptance of the underlying AlN-metal structure for the implementation of an ultrathin, high-performance uncooled resonant IR detector. The IR absorption spectra of the material stacks forming the G–AlN and reference devices were extracted from the reflectance spectra measured by FTIR in the 1–16 μm spectral range (Figure 4). The absorptance, *η*, was obtained by *η*=1−*R*−*T*, where *R* is the reflectance, and *T* is the transmittance. The transmittance here is assumed to be ~0 in the measured infrared range (1–16 μm) as the thickness (100 or 50 nm) of the underlying platinum electrode used in this work is ~3× larger than the penetration depth, *δ* (*δ*~9.46 nm at 1 μm wavelength). Whereas high reflectivity was recorded for the reference samples in the entire spectral range, three distinct absorption bands were measured for the G–AlN–Pt stack. The 3.4 μm band results from a resonance formed by the lossy AlN dielectric (***ε***_***r***_ extracted to be 3.6−j0.09) and the bottom metal electrode as confirmed by finite integration technique (FIT) simulation in the software computer simulation technology. The 11.3 μm and 15.5 μm peaks correspond to two intrinsic vibrational bands of the AlN^35^ (which are not modeled in the FIT simulation). As shown in Figure 4, >100× improvement in absorptance, *η*, at 3.4 μm, and ~10× improvement at 5 μm were recorded for the G–AlN devices compared with the reference devices employing a highly reflective top gold electrode.

**Figure 5:**
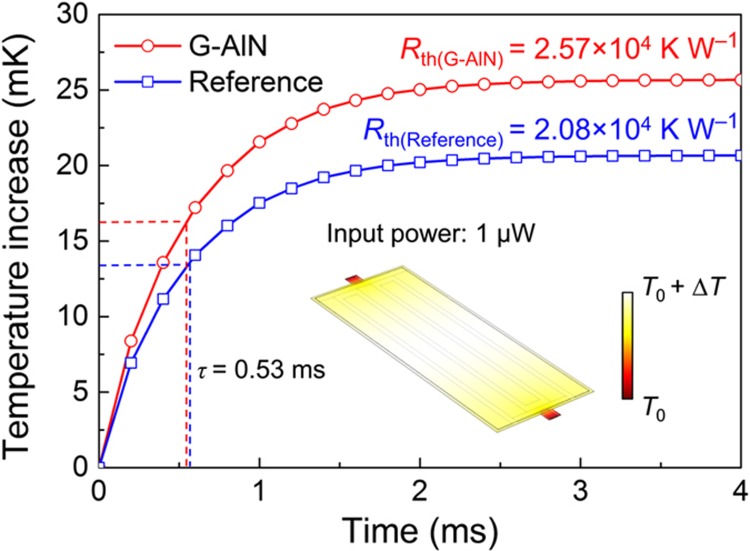
The thermal properties of the G–AlN resonator and reference device evaluated by 3D FEM simulations with applied IR power of 1 μW. The inset shows the simulated temperature distribution of the G–AlN resonator.

As indicated in Equation (2), except for the IR absorptance, the frequency response of the G–AlN NEMS resonator upon IR incident also depends on the thermal resistance and temperature sensitivity of the device. The thermal properties of the G–AlN and reference devices were evaluated by 3D FEM simulations in COMSOL with an applied IR power of 1 μW as shown in Figure 5. An increase in thermal resistance, *R*_th_, and reduction in thermal capacitance, *C*_th_, are recorded for the G–AlN resonators compared with reference devices due to the lower volume of the structure (although *R*_th_ is still primarily determined by the dimensions and material stack of the device anchors^16^, which are not optimized in this work). A low thermal time constant (~0.53 ms) is achieved for both the G–AlN and reference devices thanks to the ultrathin AlN films (460 nm) employed for the piezoelectric actuation. The temperature coefficients of frequency, TCF, of the G–AlN resonators and reference devices were measured and found to be −26.9 p.p.m. per K and –29.4 p.p.m. per K, respectively, owing to the relatively large temperature coefficient of Young’s modulus of the Au top electrode^12^.

**Figure 6:**
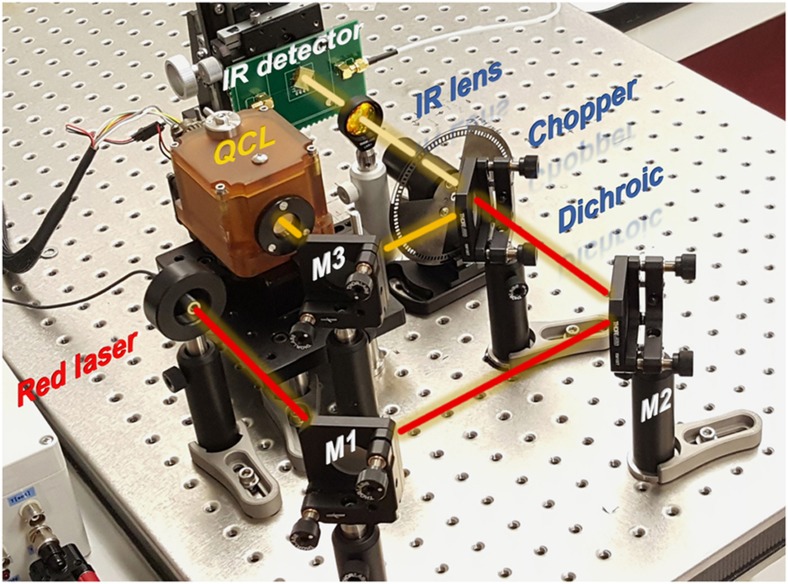
Experimental setup for the IR sensing measurement. Three reflective mirrors (M1, M2, and M3) and a dichroic filter are properly set up to co-align a red laser beam with a 5-μm QCL beam to facilitate the alignment between the QCL beam and the device under test.

The experimental setup shown in Figure 6 was used to characterize the IR detection capabilities of the fabricated G–AlN and reference devices. A 5-μm QCL, modulated at 1 Hz by a chopper and focused by a lens, was used as an IR source. Three reflective mirrors and a dichroic filter were properly set up to co-align a visible light beam from a red laser with the invisible QCL beam to facilitate the alignment between the QCL beam and the device under test. The focused beam spot in the testing plane was measured to be ~1 mm in diameter with a surface power density ~180 mW mm^−2^ at the center (see Supplementary Information). The G–AlN resonator and the reference device shown in Figure 3a were selected for this IR-sensing experiment because of their high electromechanical performance and spurious-free response near the resonance frequency. Each device was wire-bonded to a printed circuit board and exposed to the modulated IR radiation. The QCL beam was properly aligned to maximize the response of each device. The frequency response of the device under test, upon exposure to the modulated IR radiation, was continuously monitored using a network analyzer^36^.

**Figure 7:**
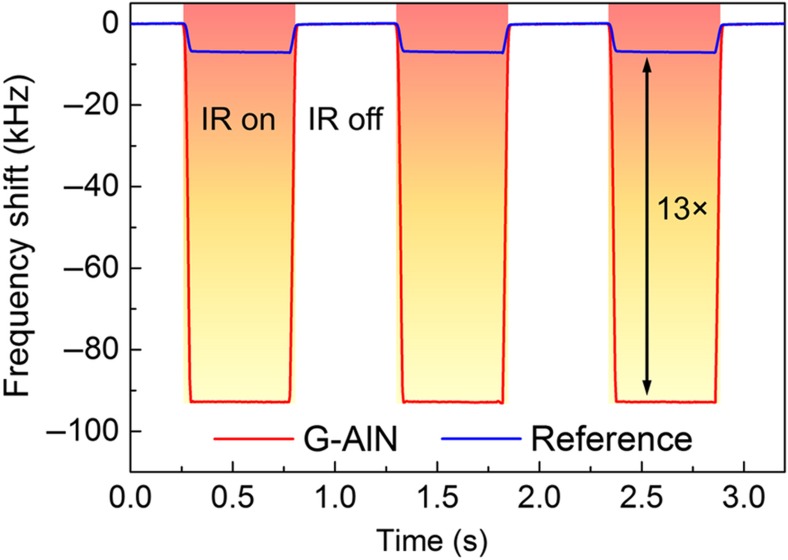
Measured frequency response of the G–AlN and reference devices exposed to a 5-μm IR radiation modulated at 1 Hz by a chopper. The G–AlN detector showed a responsivity ~13× stronger than the reference device with 100 nm Au as the top electrode.

Thanks to the largely enhanced IR absorptance at a spectral wavelength of 5 μm and the improved thermal resistance, the G–AlN detector showed a responsivity more than one order of magnitude (~13×) stronger than the reference device with 100 nm Au as the top electrode (Figure 7). This improvement in responsivity is slightly smaller than the value (~16×) estimated with Equation (2), which we attribute to the partial coverage of the bottom Pt electrode (interdigitated configuration with ~80% coverage. The frequency noise spectral density *f*_n_ of the G–AlN detector was extracted by monitoring the short-term frequency instability: the peak-to-peak frequency fluctuation of the resonance frequency when the device was not illuminated by IR was measured to be 0.1 kHz. The root mean square (r.m.s.) noise was then calculated by dividing the peak-to-peak frequency fluctuation by 6.6 for a 99.9% confidence and found to be 15 Hz. Finally, the frequency noise spectral density was calculated by dividing the r.m.s. frequency noise by the square root of the measurement bandwidth (200 Hz) and found to be *f*_n_~1.1 Hz Hz^−1/2^. Therefore, the noise equivalent power (NEP) of the detector (crucial performance metric parameter for IR detectors) was readily extracted by dividing the measured noise-induced frequency fluctuation *f*_n_ by the responsivity of the detector (assuming 11% absorption at 5 μm) and found to be ~47 nW Hz^−1/2^. The NEP at the 3.4-μm absorption peak (assuming 26% absorption) was instead estimated to be ~20 nW Hz^−1/2^.

## Discussion

**Figure 8:**
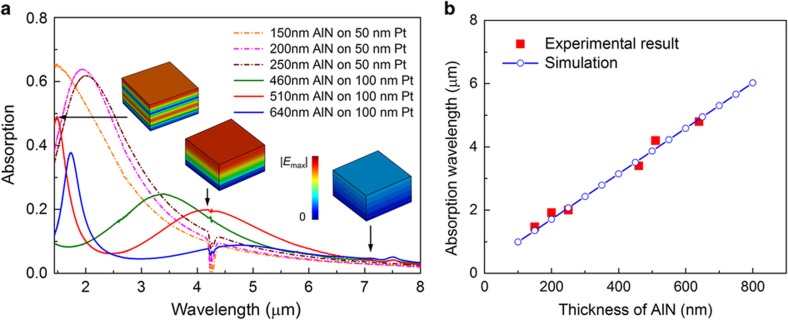
Absorption wavelength dependence on AlN thickness. (**a**) IR absorption spectra of various AlN thicknesses measured by FTIR. The insets from left to right show the simulated electric field distribution of the higher-order mode and the fundamental mode of the Fabry–Perot resonance, as well as a case of out-of-resonance for 510-nm thick AlN on 100 nm Pt, respectively. (**b**) Measured and simulated absorption wavelengths of various AlN thicknesses.

The NEP can be considered the most important performance metric for an infrared detector, and the value extracted for the fabricated proof-of-concept G–AlN IR detector proposed here is already close to the best commercially available uncooled broadband thermal detectors. Moreover, because the absorption peak depends on the thickness of the AlN plate, the proposed G–AlN technology can potentially provide unique spectral selectivity in the short-wavelength IR (SWIR) band with largely enhanced absorptance. In Figure 8a, we experimentally show that when the thickness of AlN is scaled down to and below 250 nm, the fundamental Fabry–Perot resonance shifts to a shorter wavelength (~2 μm), and the amplitude of the absorption peak increases to 60% and higher. As mentioned previously, scaling of the device thickness inevitably yields a lower device thermal mass (faster response time) and higher thermal sensitivity (higher thermal resistance), which indicates that the NEP can be improved by a factor of >6 at SWIR wavelengths compared with the one recorded at 5 μm. On the other hand, for AlN thicker than 500 nm, in which highly oriented crystals can be readily achieved, there is a higher-order mode with a strong absorption of ~50% at 1.5 μm, which will be worth exploring in the future. Figure 8b shows that the measured wavelengths at which maximum absorption is achieved for different thicknesses of AlN (fundamental mode) match closely those predicted by the FIT simulations, which highlights the capability of designing for different absorption bands by tailoring the thickness of the AlN plate. It is worth noting that the monolayer graphene sheet synthesized in this work has a relatively high sheet resistance of approximately 1–2 kΩ sq^−1^. This value could be reduced, by chemical doping or simply stacking few graphene layers, to ~380 Ω sq^−1^, enabling the implementation of a Salisbury screen featuring nearly 100% absorption at IR wavelengths^37^.

In addition to increasing the IR absorption, there is plenty of room to improve the performance of such NEMS resonant IR detectors. First, novel designs for the device anchors^16^ and vacuum package could be employed to further improve the thermal resistance up to ~10^7^ K W^−1^. Second, in order to reach the thermal fluctuation noise limit, all noise sources contributing to the generation of frequency fluctuations, such as the resonator flicker noise, random walk and drifts, need to be carefully investigated and mitigated. As a result, we expect this G–AlN technology to achieve NEP in the order of ~1 pW Hz^−1/2^, thus enabling the implementation of multispectral thermal imagers with a noise equivalent temperature difference as low as ~1 mK.

## Conclusion

In this paper, a high-frequency (307 MHz) G–AlN NEMS resonant IR detector was designed, fabricated and tested. We demonstrate that the use of a virtually massless graphene electrode not only boosts the operating frequency of the resonator but also enables the achievement of higher electromechanical performance (over 50% improved *f·Q*) compared with conventional devices employing metal electrodes. Moreover, the intrinsic IR absorption capabilities of such G–AlN nanoplates are investigated for the first time and exploited for the first experimental demonstration of a piezoelectric nanoelectromechanical resonant IR detector with enhanced absorptance (10× improvement at 5 μm and potentially >100× improvement at 3.4 μm) in a reduced volume. The achievement of high IR absorptance, in nanomechanical resonant structures with reduced volume (and hence higher thermal resistance and lower thermal capacitance) and improved electromechanical performance (*f·Q*), addresses one of the most fundamental challenges in the NEMS field and can potentially lead to the development of spectrally selective, fast (approximately tens to hundreds of microseconds) and high-resolution (noise equivalent power ~1 pW Hz^−1/2^, noise equivalent temperature difference ~1 mK) uncooled IR detectors.
