# Supplementary material for: Graphene–aluminum nitride NEMS resonant infrared detector
Source: Microsyst Nanoeng. 2016 Jun 20;2:16026. doi: 10.1038/micronano.2016.26 (PMC6444720; doi:10.1038/micronano.2016.26)
Supplement: Supplementary Information [file micronano201626-s1.pdf]

Supplementary file

Graphene–aluminum nitride NEMS resonant infrared detector

Zhenyun Qian[1], Yu Hui[1], Fangze Liu[2], Sungho Kang[1], Swastik Kar[2] and Matteo Rinaldi[1]

Microsystems & Nanoengineering (2016) **2,** 16026; doi:10.1038/micronano.2016.26; Published online: 20 June 2016

DEVICE FABRICATION PROCESS

The aluminum nitride (AlN) NEMS resonators presented in this work were fabricated using a combination of bottom-up chemical vapor deposition (CVD) growth for graphene and top-down 5-mask microfabrication for platinum (Pt), AlN, and gold (Au) layers (Figure 2 in main text).

Graphene was grown by CVD method similar to the one introduced by Li *et al*[1]. 25 μm copper (Cu) foil (item No.46986 from Alfa Aesar, 26 Parkridge Rd Ward Hill, MA 01835, USA) was cleaned in diluted nitric acid prior to growth. Then it was annealed at 1000 °C in a tube furnace with a hydrogen flow of 5 sccm for a 30 min period. After that, a 30 min methane flow of 35 sccm was used for growth while keeping the same hydrogen flow rate. After the growth, the furnace was opened and cooled down naturally at a rate of ~ 30 °C/min. To transfer graphene, the graphene-Cu foil was spin-coated with poly (methyl methacrylate) (PMMA) in order to protect and support the graphene sheet while the Cu foil was etched away in diluted nitric acid leaving only the PMMA/graphene film floating on the solution. The film was rinsed in deionized (DI) water to remove any possible residue from the etching process.

A high resistivity ($> 10^4$ Ω·cm) Silicon (Si) 4-inch wafer was used as a substrate for the fabrication of AlN NEMS resonators. A 100 nm thick Pt film (with 5 nm thick Titanium adhesive layer) was sputter-deposited and patterned by lift-off on top of the Si substrate to define the bottom IDE. Then, a 460 nm AlN film was sputter-deposited and then etched by 85% H_3PO_4 at 150 °C to access the bottom Pt electrode. The shape of the resonant plate was defined by inductively coupled plasma (ICP) etching in Cl_2 based chemistry. Then, a 100 nm thick Au film (with 5 nm thick titanium adhesive layer) was sputter-deposited and patterned by lift-off to form the probing pads. After that, the wafer was diced into 1.5 × 1.5 cm chips to enable the use of different materials (graphene, 100 nm and 50 nm Au) for the fabrication of the top electrode. The previously described graphene/PMMA sheet was rinsed in DI water and placed on top of the pre-fabricated AlN NEMS die. The transfer of the graphene layer on the AlN NEMS die was completed by dissolving the PMMA in hot acetone. The graphene layer transferred on top of the AlN NEMS die was patterned by oxygen plasma using a bilayer of PMMA and photoresist as the mask (Figure S1). Then the photoresist was exposed again and removed by developer CD30. The remaining PMMA served as a protective layer to avoid unintentional doping of the graphene layer during the following final release step in XeF_2. Finally, the graphene-AlN NEMS structure was released from the substrate by XeF_2 isotropic etching of Silicon and, after that, the PMMA protective layer was removed using warm PG remover.

High quality graphene was maintained throughout the fabrication process as confirmed by Raman spectrum taken after release of the G-AlN resonators (Figure S2). On AlN substrate, the G band locates at 1586 cm^{-1} with a full width at half maximum (FWHM) of

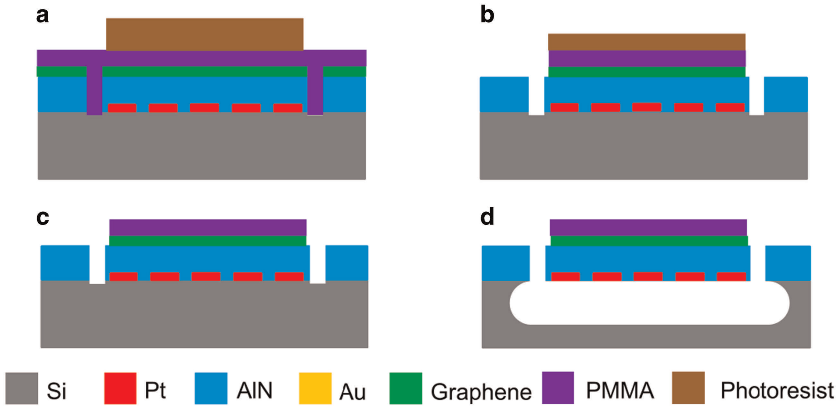

Figure S1 Graphene patterning and protection. (**a**) A 100-nm thick PMMA was spin-coated after graphene transfer, and then a layer of photoresist S1818 was spin-coated and patterned by standard photolithography; (**b**) Both PMMA and graphene was patterned by oxygen plasma using an ICP; (**c**) PR was exposed again and removed by developer CD 30; (**d**) With the PMMA protective layer, the graphene-AlN NEMS structure was released from the substrate by XeF_2 isotropic etching of Silicon.

[1]Department of Electrical and Computer Engineering, Northeastern University, Boston, MA 02115, USA and [2]Department of Physics, Northeastern University, Boston, MA 02115, USA.
Correspondence: Matteo Rinaldi (rinaldi@ece.neu.edu)

22 cm^{-1}. The G′ band locates at 2700 cm^{-1} with an FWHM of 40 cm^{-1}. The G-to-G′ ratio is ~0.5. The D band is negligible. All these features indicate that the graphene is high quality monolayer.

The fabrication of the reference device chips was instead completed by depositing and patterning a 100-nm thick Au layer (instead of graphene) to form the device top electrode (gold is typically used as top metal electrode in NEMS resonant sensors since it can be easily functionalized with thiolated ligands[2]). The Au top-electrode was deposited by e-beam evaporator and patterned by lift-off. Reference chips were released from the substrate by XeF_2 isotropic etching of Silicon without protection using same recipe as that of G-AlN chips.

FEM SIMULATIONS

FEM simulations were performed with COMSOL Multiphysics Modeling Software. The 3D and 2D resonator models were built based on the actual devices' lateral geometries set at design level and vertical dimensions from the measured thickness of the deposited Pt, AlN, and Au (for reference device only) layers. The

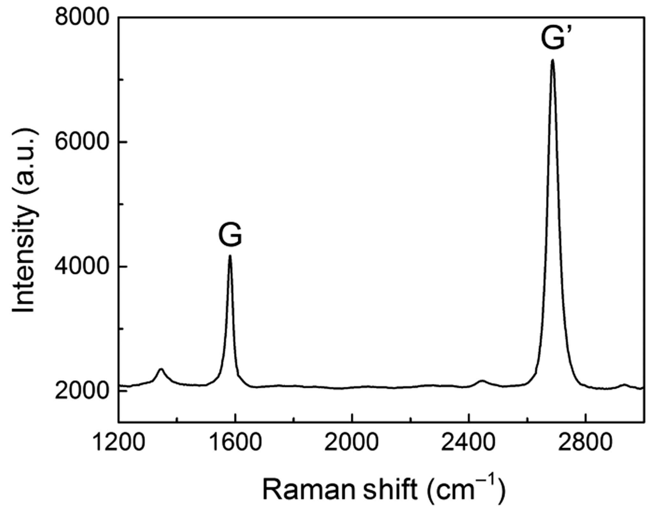

Figure S2 Typical Raman spectrum of graphene taken on the fabricated G-AlN NEMS resonator after release process.

default material properties values defined in COMSOL (density, Young's modulus, Poisson's ratio, and piezoelectric coefficients (for AlN only)) were used in the simulations, with the exception of the relative permittivity of the sputtered AlN thin film which was manually set to be 9.0 based on previous measurements. A 3D simulation of a reference device with 100 nm thick Au top-electrode and a pitch size of 15 μm was first performed for as shown in Figure S3a. The top electrode was set to be at a floating potential with zero charge, while the metal fingers forming the bottom IDE were set to be ground and terminal (with a 1 V electric potential) with an alternated arrangement. Using the COMSOL's physics model of piezoelectric devices, the device resonance frequency was estimated (by frequency domain simulation) to be 229.4 MHz in good agreement with the ~226.9 MHz average resonance frequency value obtained experimentally from 15 fabricated devices (based on the simulated design). Given the relatively high computational complexity of 3D simulations, we simplified the FEM model to 2-dimensions (device cross-section) and simulated its resonance frequency (~20× faster computation time than the 3D case) obtaining a value of ~228.5 MHz (Figure S3b,c), in excellent agreement with the one estimated by 3D simulations. Therefore, the FEM simulations of the multiple designs investigated in this work were performed using a 2D model. For the FEM simulations of the graphene-electrode devices, the entire top surface of the AlN nano plate was set to be at a floating potential with zero charge to emulate an 'ideal top-electrode' providing the necessary electronic confinement of the RF field within the AlN membrane, without adding any mechanical mass or strain that is associated with conventionally-deposited metal-electrodes. The simulations of the metal-free, all-graphene-electrode devices were performed in a similar manner, but replacing the bottom interdigitated Pt electrodes with conductive boundaries on the edge of the AlN nano plate with alternated polarity. A *Q*-factor of 1000 (matching our typical experimental results) was employed for all the simulations.

CHARACTERIZATION OF LASER BEAM SPOT

The spot size of the focused IR beam was measured at the plane where the devices were placed using the knife-edge method described by Khosrofian *et al.*[3] and found to be ~1 mm in diameter (radius ω ~0.5 mm, Figure S4). The total power P_{total} was measured by a commercial thermal power sensor (Thorlabs item No. S302C, 56 Sparta Avenue, Newton, New Jersey 07860, USA) and found to be ~70 mW. Assuming a uniform Gaussian laser

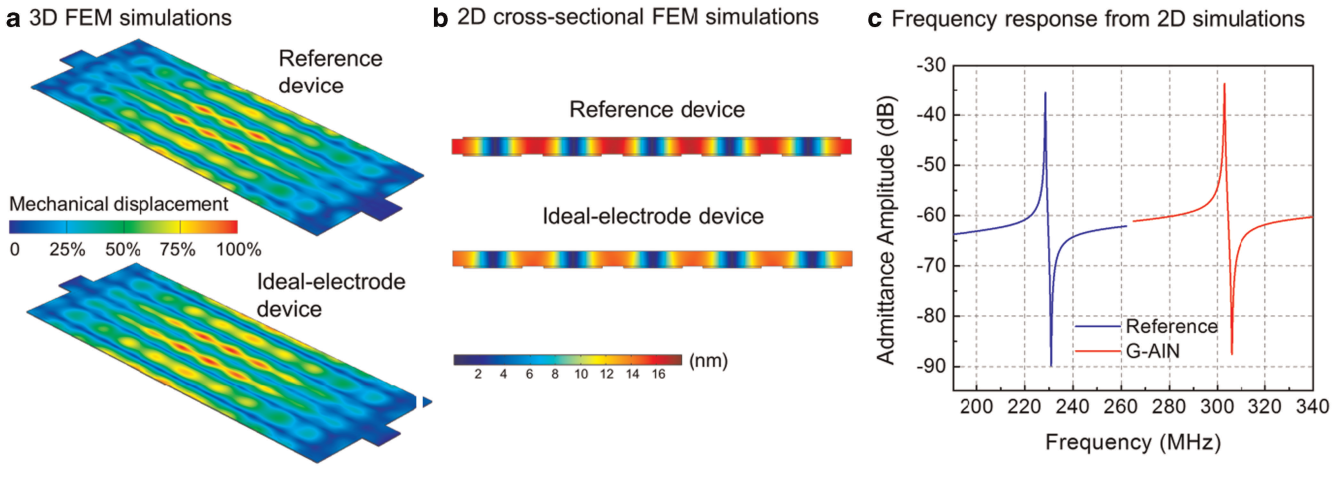

Figure S3 (**a**) Spatial distribution of the total mechanical displacement obtained from 3D FEM simulation. (**b**) Cross-sectional spatial distribution of mechanical displacement, and (**c**) Admittance curves obtained from 2D FEM simulation.

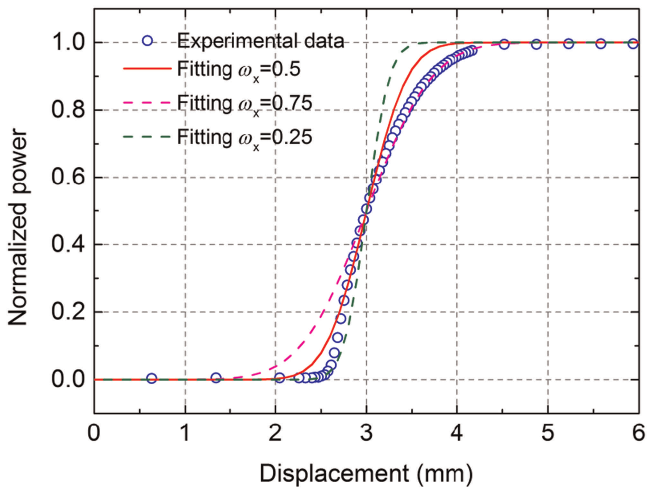

Figure S4 Knife-edge measurement on horizontal direction (x) of the focused IR beam.

beam, the surface power density I_0 was estimated to be ~180 mW mm^{-2} at the center of the beam. Considering that the device size ($A = 75$ μm × 200 μm) is much smaller than the spot size of the beam, the IR power delivered to the device was calculated to be ~$I_0 \cdot A = 2.7$ mW. However, it is worth noting that IR laser beam emitted by the un-collimated 5-μm QCL prototype (provided by Pendar Technologies, 30 Spinelli Place, Cambridge, MA 02138, USA) is not perfectly Gaussian, as shown in Figure S4, which negatively affects the accuracy of the I_0 estimation using the one-dimensional knife-edge method. In addition, the measurement uncertainty (+/−5%) from the commercial thermal power sensor further lower the accuracy of the estimated delivered power.

COMPETING INTERESTS

The authors declare no conflict of interest.

REFERENCES

1 Li X, Cai W, An J *et al.* Large-area synthesis of high-quality and uniform graphene films on copper foils. *Science* 2009; **324**: 1312–1314.

2 Rinaldi M, Zuniga C, Piazza G. ss-DNA functionalized array of AlN contour-mode NEMS resonant sensors with single CMOS multiplexed oscillator for sub-ppb detection of volatile organic chemicals. IEEE 24th International Conference on Micro Electro Mechanical Systems (MEMS 2011); 23-27 Jan 2011; Cancun, Mexico; 2011: 976-979.

3 Khosrofian John M, Garetz Bruce A. Measurement of a Gaussian laser beam diameter through the direct inversion of knife-edge data. *Applied Optics* 1983; **22**: 3406–3410.
